# RNA-Seq Comprehensive Analysis Reveals the Long Noncoding RNA Expression Profile and Coexpressed mRNA in Adult Degenerative Scoliosis

**DOI:** 10.3389/fgene.2022.902943

**Published:** 2022-08-11

**Authors:** Xin Shi, Panpan Li, Xiang Wu, Zhihua Wang, Gang Zhao, Jun Shu

**Affiliations:** ^1^ The Second Affiliated Hospital of Kunming Medical University, Kunming Medical University, Kunming, China; ^2^ Faculty of Medicine and University Hospital of Cologne, University of Cologne, Cologne, Germany

**Keywords:** adult degenerative scoliosis, long noncoding RNA, messenger RNA, whole blood, nucleus pulposus tissue, gene ontology, pathway analysis

## Abstract

**Objective:** Owing to the intensification of the aging process worldwide, the prevalence of adult degenerative scoliosis (ADS) is increasing at an alarming rate. However, genomic research related to the etiology of ADS is rarely reported worldwide. Since long noncoding RNAs (lncRNAs) play a pivotal role in the progression of human diseases, this study aimed to investigate ADS-associated messenger RNAs (mRNAs) and lncRNAs by RNA sequencing (RNA-seq), as well as performed comprehensive bioinformatics analysis based on the lncRNA–mRNA coexpression network and protein–protein interaction (PPI) network.

**Methods:** Initially, six whole blood (WB) samples were obtained from three ADS and three nondegenerative lumbar trauma patients who underwent surgical operation for RNA-seq exploration to construct differential mRNA and lncRNA expression profiles. Subsequently, quantitative RT-PCR (qRT-PCR) was performed to validate three randomly selected differentially expressed mRNAs and lncRNAs derived from the nucleus pulposus (NP) tissue of 14 other subjects (seven ADS patients and seven nondegenerative lumbar trauma patients), respectively.

**Results:** A total of 1,651 upregulated and 1,524 downregulated mRNAs and 147 upregulated and 83 downregulated lncRNAs were screened out from the RNA-Seq data, which constructed coexpression networks to investigate their regulatory interactions further. GO gene function prediction revealed that lncRNA-targeted genes might play a vital role in ADS *via* participation in multiple biological processes such as the AMPK signaling pathway, lysosomes, and ubiquitin-mediated proteolysis, as well as cellular metabolic processes. Moreover, the expression levels of three selected lncRNAs and mRNAs were validated by qRT-PCR, respectively, demonstrating that the relative expression levels were consistent with the RNA-seq data. Notably, the dysregulated RNAs, *AKT1*, *UBA52*, *PTPN12*, and *CLEC16A*, were significantly differentially expressed in ADS WB samples and might serve as potentially regulated genes for research in the future.

**Conclusions:** This study provides the first insight into the altered transcriptome profile of long-stranded noncoding RNAs associated with ADS, which paves the way for further exploration of the clinical biomarkers and molecular regulatory mechanisms for this poorly understood degenerative disease. However, the detailed biological mechanisms underlying these candidate lncRNAs in ADS necessitate further elucidation in future studies.

## Introduction

Adult degenerative scoliosis (ADS) is defined as a three-dimensional spinal deformity with a Cobb angle >10° in the coronal plane (Aebi, 2005), which refers to the structural curve formed by the previously normal spine after skeleton maturity; hence, it is also described as new-onset adult scoliosis (Grubb et al., 1988). Because of the increase in age and the aggravation of degeneration, up to 90% of patients with ADS may develop central spinal stenosis with neurogenic claudication, and 60%–80% suffer low back pain (Grubb et al., 1994; Silva and Lenke, 2010; Cho et al., 2014), even it is possible to cause complications such as syringomyelia and Charcot arthropathy of the lower limbs (Shi et al., 2021), which seriously affects the patient’s physical and mental health. According to current statistics, approximately 8.9% of people have been found to have ADS among the 40-year-old age group, with a significantly increased risk of scoliosis from 50 to 60 years. However, due to the complicated pathogenesis of ADS, its etiology is not completely clear. Comprehensive studies suggest that ADS is the consequence of a complex interaction between asymmetric intervertebral disc degeneration (IDD), intervertebral facet joint overload, lifestyle factors (smoking, obesity, etc.), and genetic factors (Vernon-Roberts et al., 2008; Silva and Lenke, 2010; York and Kim, 2017). It is estimated that by 2050, the proportion of the world’s population over 60 years old will nearly double (Schwab et al., 2005). At the same time, coupled with the acceleration of age-related spinal degeneration, the prevalence of ADS is increasing at an alarming rate (Ploumis et al., 2007), which is bound to substantially increase the economic burden on individuals and society from a public health perspective.

Long noncoding RNAs (lncRNAs) refer to RNA transcripts with a length of more than 200 nucleotides and lack protein-coding capabilities (Ponting et al., 2009; Li et al., 2016; Ren et al., 2018). An increasing body of evidence suggests that lncRNAs are involved in various processes of cellular activities, such as adipogenesis, apoptosis, pyrolysis, cell differentiation, epigenetic modification, and tumorigenesis and regulation (Fang and Fullwood, 2016; Geng and Tan, 2016; Bach and Lee, 2018; Ma et al., 2018; Tang et al., 2019). lncRNAs are implicated in the onset and development of various multifactorial diseases such as cancer, cardiovascular diseases, autoimmune diseases, and neurodegenerative diseases (Huang et al., 2013). Furthermore, lncRNAs can exist as a stable form in tissues and body fluids as immunity to endogenous RNase activity (Huang et al., 2016). Recently, some studies have illustrated that lncRNAs can facilitate autophagy and apoptosis of nucleus pulposus (NP) cells involving IDD. Chen et al. identified that overexpression of lncRNA XIST inactivates the PI3k/Akt signaling pathway to regulate autophagy of NP cells in IDD (Chen et al., 2021). Sun et al. demonstrated that lncRNA H19 promotes autophagy and apoptosis of NP cells through miR-139-3p/CXCR4/NF-kappa B axis to exacerbate IDD (Sun et al., 2021). Currently, the genomic studies related to the etiological mechanism of ADS have been reported rarely worldwide. We chose whole blood(WB) for the analysis because it has proven to be a useful surrogate of gene expression in the peripheral and central nervous system and can be collected in a minimally invasive manner that is amenable for potential future diagnostic test development (Parisien et al., 2017). Therefore, in this study, high-throughput sequencing was carried out to examine the lncRNA and mRNA expression profiles in WB samples collected from ADS patients and nondegenerative lumbar trauma patients, and verified in the NP tissues, which will fill in the gaps in the etiology of ADS and provide a research basis for the identification of causative genes and the selection of targeted therapeutic candidates in the future.

## Materials and Methods

### Ethical Approval and Patient Consent

This study was approved by the Ethics Review Committee of the Second Affiliated Hospital of Kunming Medical University. The study was complied with the “Declaration of Helsinki” (revised in 2013). All human tissues were obtained and utilized with the informed consent of the participants. All samples were collected at the Second Affiliated Hospital of Kunming Medical University from January 2017 to December 2018.

### Participants and Sample Collection

WB sample and NP tissue was obtained from 10 patients with ADS who underwent surgical operation (ADS group). The inclusion and exclusion criteria for the ADS group are illustrated in Table 1, Table 2: age range: 45–59 years (mean age: 53.4 ± 4.84 years) and body mass index (BMI)18.5 ≤ BMI<24. The patient was finally diagnosed as ADS upon a comprehensive assessment based on the medical history, clinical physical examination, and radiographic examination (X-rays show that the lumbar vertebrae as the apex with a Cobb angle range of coronal scoliosis are ≥15°; MRI indicates varying degrees of degeneration of the IVD, intervertebral facet joints, and ligamentum flavum).

**TABLE 1:** Inclusion criteria of the ADS group.

Inclusion criteria of the ADS group
(1) According to the medical history, clinical examination and imaging examination were diagnosed as ADS
(2) Over 50 years old
(3) 18.5 ≤ body mass index (BMI) < 24
(4) With the lumbar spine as the vertex, X-ray showed that the Cobb Angle range of scoliosis on the coronal plane was greater than or equal to 15°
(5) CT and MRI suggested different degrees of degeneration of intervertebral discs, intervertebral facet joints, ligamentum flavum, etc.

**TABLE 2:** Exclusion criteria of the ADS group.

Exclusion criteria of the ADS group
(1) Medical history of metabolic bone disease, spinal trauma, and spinal infection
(2) History of spinal surgery
(3) History of autoimmune diseases, systemic inflammatory diseases, solid tumors, or hematological malignancies
(4) Complicated with severe osteoporosis and severe liver or kidney insufficiency
(5) Pregnant or lactating women
(6) Scoliosis secondary to other organic spinal lesions, such as tumor, trauma, *tuberculosis*, and metabolism
(7) History of lumbar spine surgery, congenital scoliosis, or undetected idiopathic spinal column scoliosis in adolescents

Meanwhile, we recruited 10 lumbar spine trauma patients without disc degeneration (Normal group) who underwent the lumbar surgical operation (the inclusion criteria and exclusion criteria of the Normal group are shown in Table 3 and Table 4), and obtained normal NP tissues and WB samples, age between 18 and 35 years (mean age: 24 ± 3.94 years), 18.5 ≤ body mass index (BMI) < 24.

**TABLE 3:** Inclusion criteria of the normal group.

Inclusion criteria of the normal group
(1) Age between 18 and 35 years
(2) 18.5 ≤ BMI<24
(3) After X-ray, CT, and MRI examination, there is no spine-related disease, intervertebral disc and facet joint structure is complete, and there is no lesion
(4) CT and MRI suggested different degrees of degeneration of intervertebral discs, intervertebral facet joints, ligamentum flavum, etc.

**TABLE 4:** Exclusion criteria of the normal group.

Exclusion criteria of the normal group
(1) Medical history of autoimmune diseases, systemic inflammatory diseases, solid tumors, or hematological malignancies
(2) Complicated with severe osteoporosis, severe liver, and kidney dysfunction
(3) Pregnant or lactating women

Five milliliters of fasting WB samples were collected from the median cubital vein of each participant between 9:00 and 9:30 AM. All WB samples were incubated in a PAXgene Blood RNA tube (BD, USA) for 24 h at −20 °C and then transfer it to a −80°C refrigerator for preservation. Then transfer it to a −80°C refrigerator for storage. After collecting all samples, place whole blood RNA tubes on a metal rack and thaw at room temperature (18°C-25°C) for two hours. After thawing, carefully invert the whole blood RNA tube ten times. Finally, total RNA extraction, detection, lncRNA library construction, and sequencing were performed on six samples. Eventually, the NP tissues were isolated from each participant during the operation and temporarily stored in cold phosphate-buffered saline (PBS) and then quickly transferred to the laboratory. After, the samples were washed in PBS to remove all blood and annulus fibrous tissue and then frozen at −80°C for further examination.

### RNA Extraction and Quality Control

**FIGURE 1:**
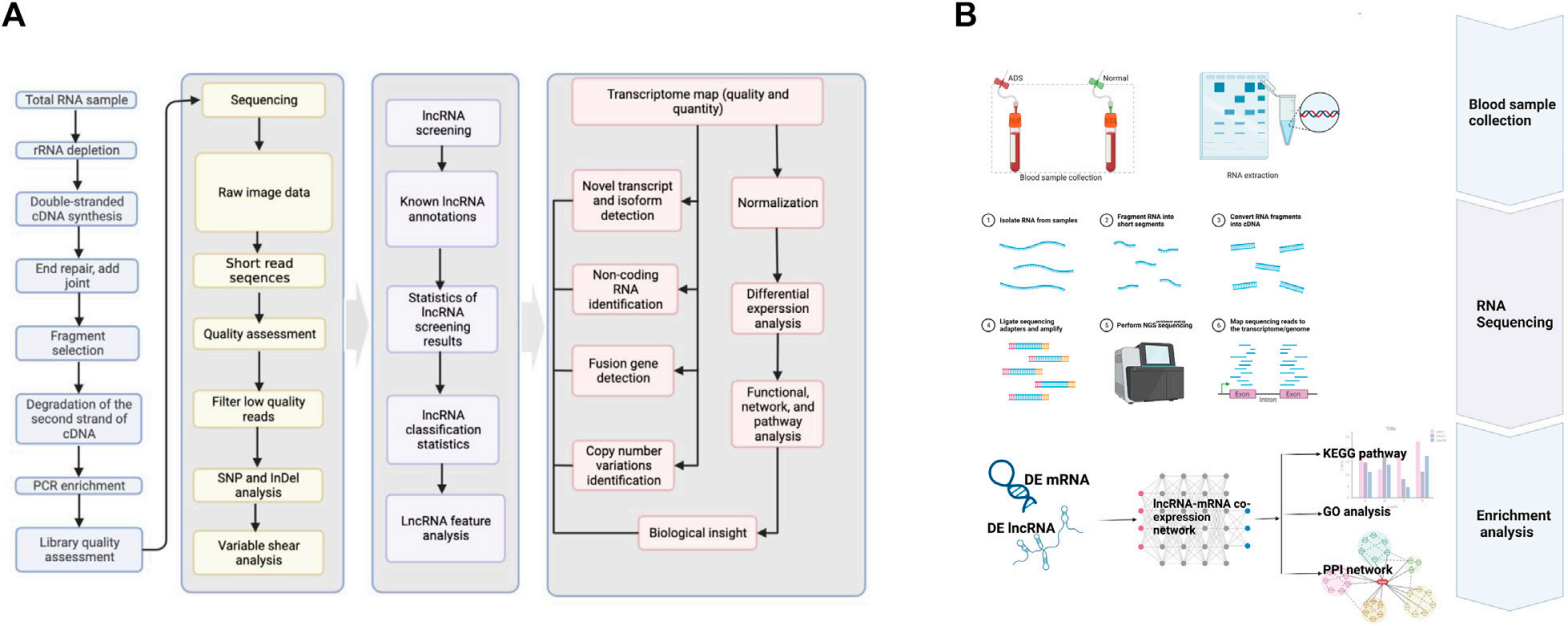
**(A)** RNA-seq bioinformatic pipeline. **(B)** Schematic diagram of the workflow of this study.

According to the manufacturer’s instructions, the total RNA was extracted from each WB sample employing TRIzol reagent (Invitrogen, Carlsbad, CA, United States). RNA purity was determined by using a spectrophotometer (NanoDrop-1000, Thermo Fisher Scientific) with OD260/OD280 readings (10mM Tris, pH 7.5) between 1.8 and 2.1. The temperature was maintained at −80°C, while the RNA quality was measured by the Agilent 2,100 TapeStation (Agilent Technologies, United States) Biochip Analysis System. Eventually, samples were selected with RNA integrity values > 7.0 to construct cDNA libraries. The RNA-seq bioinformatics pipeline is illustrated in Figure 1A, and the workflow for this study is schematically shown in Figure 1B.

### Construction of cDNA Libraries

The Illumina^®^ NEBNext^®^ Ultra™ Directed RNA Library Preparation Kit is utilized to construct Illumina True-Seq strand lncRNA libraries. In brief, the lncRNA library is constructed as a strand-specific library. The first strand of cDNA is synthesized by reverse transcription in the same method as the conventional NEB library build. Distinctively, the dTTP in the dNTPs was substituted by dUTP when the second strand was synthesized, which was followed by cDNA end repair, the addition of A-tails, ligation of sequencing junctions, and length screening in the same manner. Then, the second strand of the U-containing cDNA was preferentially degraded using USERase, as well as purification and enrichment of the product by PCR to create the final cDNA library, which was quantified *via* Agilent 2,200. Ultimately, six cDNA libraries were constructed for this study.

### RNA Sequencing

Mapping of paired-end reads: prior to reading the mapping, clean reads, reads with >5% ambiguous bases (denoted as N), and low-quality reads containing >20% of bases with mass <20 are obtained from the original read by removing the splice sequence. The filtered reads were aligned to the human genome [version: GRCh38 National Center for Biotechnology Information (NCBI)] by applying HISAT2. Typically, the percentage of reads generated by sequencing should be above 70% (Total Mapped Reads or Fragments), as long as the reference genome is selected appropriately and the experiment is free from contamination. Then, the HTSeq software was utilized to obtain gene count, which was quantitatively analyzed for each sample of the species based on the known genotype. Finally, the RPKM method was employed to determine gene expression, which was obtained from the samples based on expression statistics for various gene types.

### Identification of Differentially Expressed lncRNAs and mRNAs

Sequence reads were matched by the TopHat 2.0 program (Vernon-Roberts et al., 2008) to obtain comparison files. Annotated references for mRNA and lncRNA analysis were derived from the RefSeq and Ensembl transcriptional databases. Reference genomes of reads were filtered through HISAT2 alignment analysis. Cuffmerge software was applied to screen lncRNAs. The gene expression data of six samples were obtained through the fragments per kilobase of exon model per mapped reads (FPKM), the methods for standardization of gene expression levels. In this analysis, to enhance the reliability of the analytical algorithm results, differentially expressed (DE) mRNAs and DE lncRNAs were identified based on the FPKM value of the individual gene in each sample by edgeR (Robinson et al., 2010). The absolute logarithmic fold change (LFC) ≥ 1 (|log2 fold change|≥ 1) and *p*-value <0.05 were adopted as the screening criteria. Principal component analysis (PCA), implemented in the prcomp function of R, was conducted to abstract the main characteristics of the data, which served as an indicator of the overall state of the data.

### Construction of the lncRNA–mRNA Weighted Coexpression Network

LncRNAs play a biological role by regulating mRNAs. A coexpression network of lncRNA/mRNA was constructed to investigate the potential interactions between lncRNA and mRNA, which could identify the key lncRNAs involved in ADS and their potential functional. This study analyzed the correlation between lncRNA and mRNA in the samples using the Pearson correlation coefficient method. The absolute values of correlation coefficients >0.95 and *p* < 0.001 were specified as screening criteria. Then, the biological functions of lncRNAs were predicted by performing a functional enrichment analysis on mRNAs.

### GO and KEGG Pathway Enrichment Analyses

All DE mRNAs were subjected to GO and KEGG pathway analysis, which could investigate the potential role of lncRNAs coexpressed with the related mRNAs. GO analysis (http://www.geneontology.org) is commonly employed in large-scale gene function enrichment studies (York and Kim, 2017) to construct gene annotations. The *p*-values for DE genes are measured and corrected. Then multiple hypothesis testing is performed so that the probability of GO term being enriched by differential genes could be calculated more accurately. Typically, GO analysis covers three domains: cellular composition (CC), molecular function (MF), and biological process (BP). The Kyoto Encyclopedia of Genes and Genomes (KEGG) (https://www.genome.jp/kegg/) is a biological system database that integrates genome, chemistry, and system function information. KEGG can be used to link genomes and biological functions through PATHWAY tracking. In living organisms, different genes coordinate with each other to perform specific biological functions. The significant enrichment of pathways allows the identification of the dominant biochemical metabolic pathways and signal transduction pathways involved in a particular gene. The false discovery rate (FDR) was used to denote the significance of the *p*-value (an FDR value of <0.05 was recommended).

### Protein–Protein Interaction Network Construction and Module Selection

The target genome sequences are aligned with the protein sequences of close relatives or model species from the STRING protein interaction database (http://string-db.org/) utilizing Blastx, whereby interaction networks are constructed from the protein interaction relationships of selected close relatives or model species. Thus, the differential gene–protein interaction network data files can be imported directly into Cytoscape software for visual editing.

### Quantitative Real-Time PCR Validation

The RNA was extracted from the NP samples of additional ADS group (n = 7) and Normal group (n = 7), respectively, in which cDNA was subsequently synthesized by reverse transcription reactions. Add 1 μg of total RNA to an enzyme-free PCR tube on ice; add 5×iScript Reaction Mix (4 μl) and iScript Reverse Transcriptase (1 μl) to each well, then make up 20 μl with nuclease-free water, and mix well and centrifuge briefly (5s); Reverse transcription conditions: 37°C (60 min) −85°C (5°min) −4°C (stop); after the reaction was stopped, the cDNA was placed in a refrigerator at −20°C for PCR, and the primer was diluted with DEPC water according to the instructions; prepared to conform to the following systematic procedure: 2×iTaqTM universal SYBR Green supermix (10 μl), forward and reverse primers (1.8 μl), DNA template (1 μl), and DEPC (7.2 μl). Centrifuge briefly (5 s) after mixing. The PCR procedure is as follows: 1) 95°C (5 min); 2) 95°C (15 s) −60°C (30 s), 40 cycles; 3) termination at 4°C. The 2^-ΔΔCt^ method was employed to calculate the relative RNA expression level. This value is represented as mean ± SD. A student t-test was conducted, and when *p*-value < 0.05, the results were considered to be significantly different.

### Statistical Analysis

Statistical analyses were performed by the Statistical Package for the Social Sciences (SPSS) version 25.0 software (SPSS Inc., Chicago, IL, United States). Data are presented as the mean ± SD of the results of at least three independent experiments. Appropriately, Student t-tests and Mann–Whitney U-tests were applied to determine significant differences between groups. The Pearson correlation coefficient was applied to inspect the correlation of expression between samples. A *p*-value < 0.05 was considered statistically significant for all tests. Moreover, in order to correct the batch effect, the RUVseq package for the R language was applied for batch correction. In addition, heatmaps and volcano maps were exported from the R language Heatmap package 2, scatter maps, and PCA results from the ggplot2 package.

## Results

### Overview of Differentially Expressed lncRNAs and mRNAs

**FIGURE 2:**
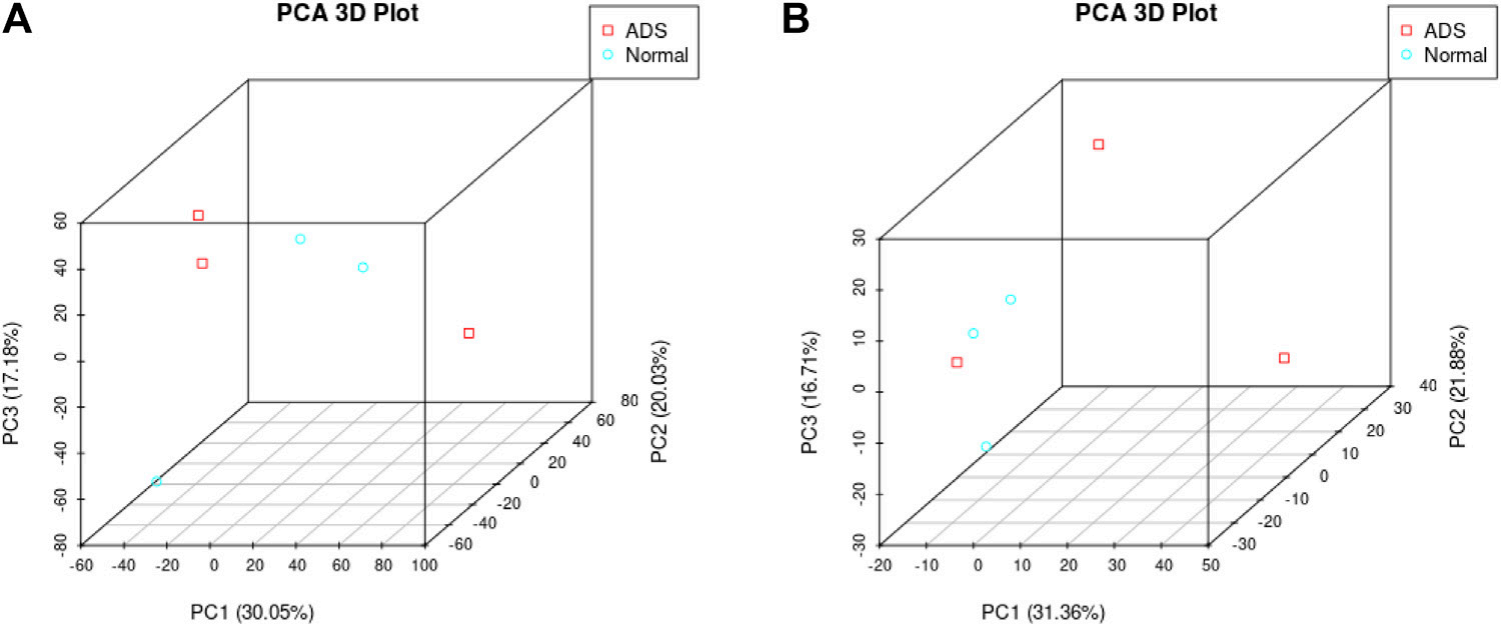
Principal component analysis (PCA) shows the clustering of mRNA and long noncoding RNA (lncRNA) expression matrices in different samples. **(A)** PCA of mRNA expression between the ADS cluster and normal cluster. The red dots represent the ADS samples, and the blue dots represent the normal tissue (Normal) samples. **(B)** PCA of the lncRNA expression between the ADS cluster and normal cluster. The red dots represent the ADS samples, and the blue dots represent Normal samples.

**FIGURE 3:**
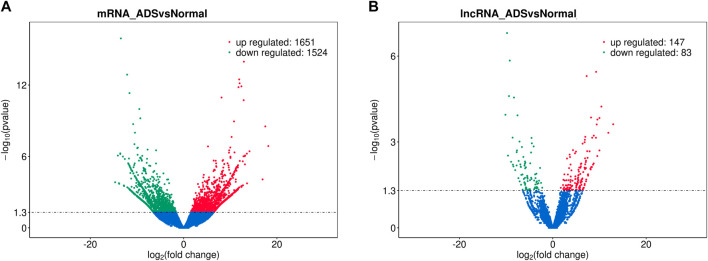
Analysis of differentially expressed mRNAs and lncRNAs. Volcano plot showing differentially expressed **(A)** mRNAs and **(B)** lncRNA in ADS and normal groups.

**FIGURE 4:**
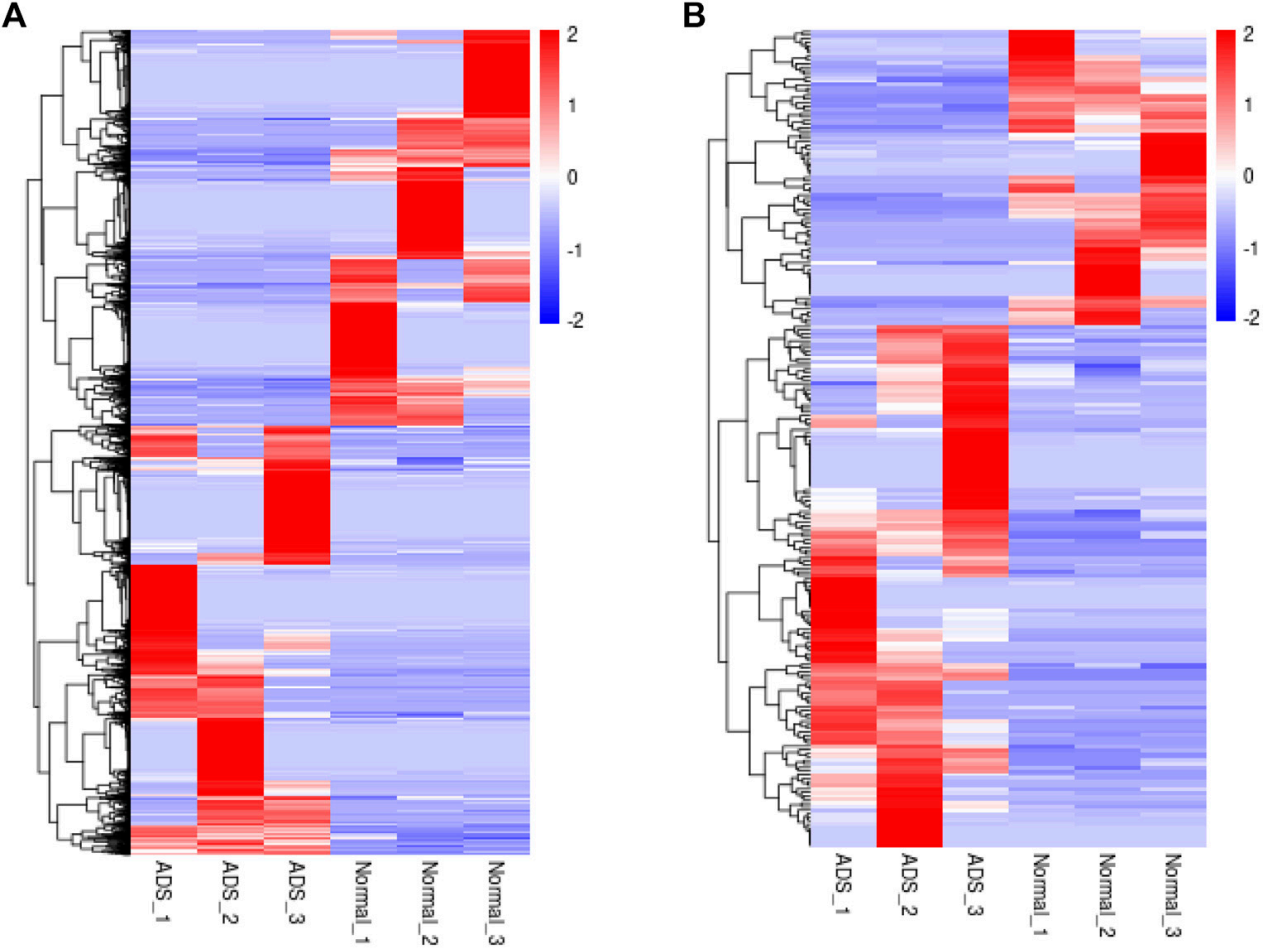
Clustering analysis. Hierarchical clustering illustrates distinguished expression difference of **(A)** mRNAs and **(B)** lncRNAs between the two groups and homogeneity between groups.

In order to determine whether there was clustering or outliers in the sample set, the differences between the clustering of the mRNA (Figure 2A), lncRNA (Figure 2B) expression matrixes of the ADS, and Normal samples in different datasets were examined using three-dimensional principal component analysis (PCA). The results showed that ADS was well distinguished from the Normal samples. The expression levels of lncRNAs and mRNAs in WB samples from 3 ADS patients and 3 normal patients were analyzed comparatively through RNA-seq. DE lncRNAs and mRNAs were screened following the criteria |log2 (fold change)| > 1, *p*-value < 0.05. *LNC_000044* (log_2_ (fold change):15.093, *p* = 2.56E-07) and *ENST00000424684.2* (log_2_ (fold change): 10.144, *p* = 9.61E-05) were the most upregulated and downregulated lncRNAs among the identified lncRNAs. We constructed volcano maps to visualize the differential expressions of mRNAs and lncRNAs between samples (Figures 3A,B). The results showed that a total of 230 lncRNAs (147 upregulated and 83 downregulated) and 3,175 mRNAs (1,651 upregulated and 1,524 downregulated) produced significant changes. *EPB41* (log_2_ (fold change):18.16001835, *p* = 2.23E-07) and *MYADM* (log_2_ (fold change): 14.67313747, *p* = 0.000185825) were the most upregulated and downregulated mRNAs among the identified mRNAs. The top 10 most DE mRNAs and lncRNAs (five upregulated and five downregulated) are displayed, respectively, in Table 5 and Table 6. Furthermore, heatmaps were created to group lncRNAs and mRNAs at the expression level between samples (Figures 4A,B).

**TABLE 5:** Top 10 differentially expressed mRNAs.

mRNA name	Gene name	Log_2_ (fold change)	*p*-value	Regulated
*ENST00000649717*	*EPB41*	18.16001835	2.23E-07	Up
*ENST00000642937*	*EPB41*	17.51348319	5.79E-09	Up
*ENST00000370857*	*MBNL3*	16.92620439	0.000107137	Up
*ENST00000413219*	*SDCBP*	14.12285154	6.45E-07	Up
*ENST00000367051*	*CR1*	13.59940505	0.000229326	Up
*ENST00000336967*	*MYADM*	−14.67313747	0.000185825	Down
*ENST00000394419*	*ACTN1*	−14.08400562	1.37E-06	Down
*ENST00000518721*	*ASAP1*	−13.85480643	0.000220224	Down
*ENST00000513163*	*FBXL5*	−13.8071376	0.000248399	Down
*ENST00000371706*	*SEC16A*	−13.57253774	9.53E-07	Down

**TABLE 6:** Top 10 differentially expressed lncRNAs.

lncRNA name	Gene name	Log_2_ (fold change)	*p*-value	Regulated
*LNC_000044*		15.093	2.56E-07	Up
*LNC_000009*		12.943	0.00029719	Up
*LNC_001060*		11.905	0.00057081	Up
*LNC_006155*		10.413	4.82E-05	Up
*LNC_005341*		9.999	0.000127804	Up
*ENST00000424684.2*	*RP11-403I13.7*	−10.144	9.61E-05	Down
*ENST00000627173.1*	*LINC00891*	−9.798	9.72E-08	Down
*LNC_006024*		−9.593	0.00290163	Down
*LNC_000026*		−9.383	2.54E-05	Down
*ENST00000592135.5*	*CTD-3014M21.4*	−9.212	1.19E-06	Down

### Functional Analysis of Differentially Expressed mRNAs

**FIGURE 5:**
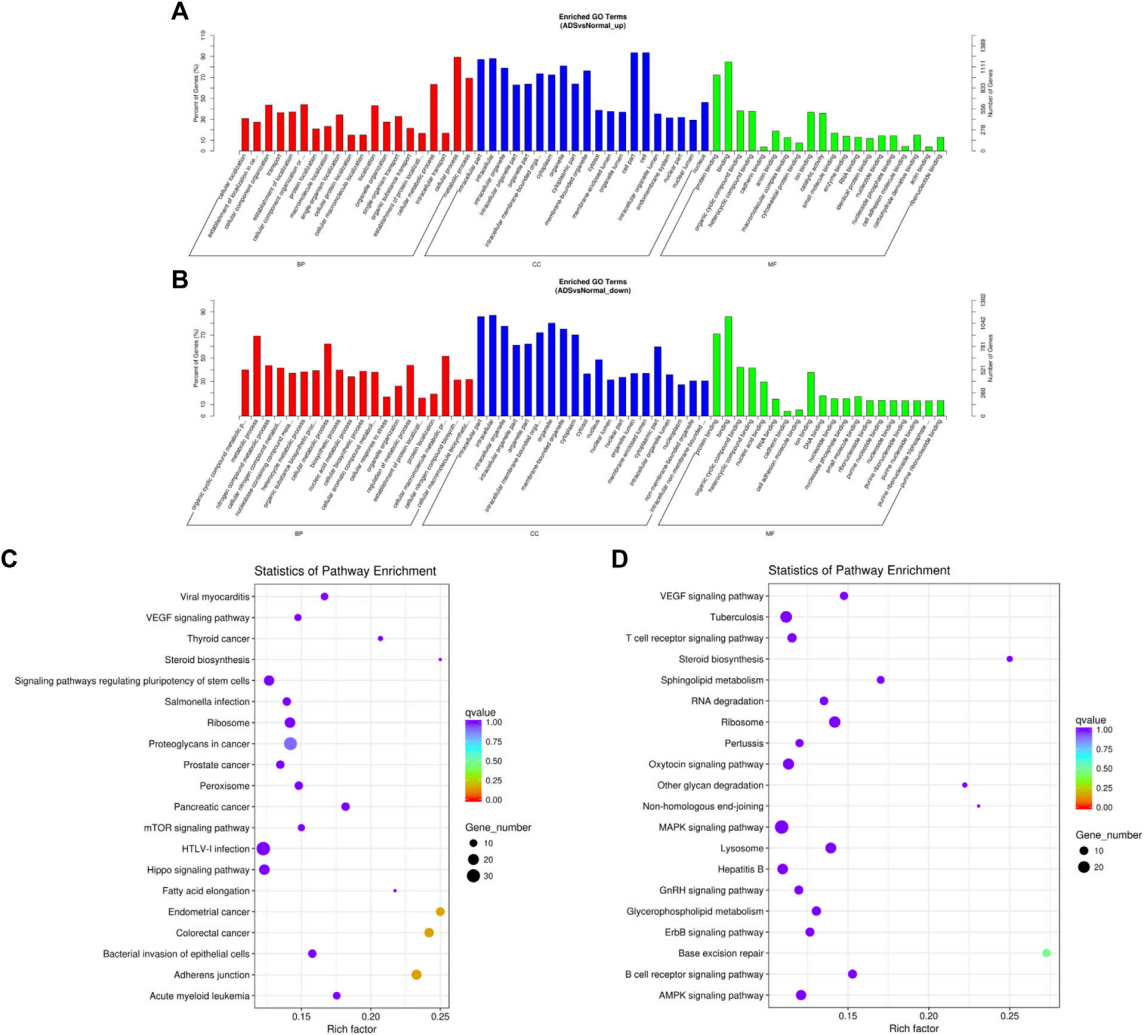
Comparison of functional annotations for differentially expressed mRNAs. The 20 most enriched Gene Ontology (GO) terms for the parental genes of **(A)** the upregulated differentially expressed mRNA and **(B)** the downregulated differentially expressed mRNA. Enriched GO terms are on the vertical axis, and the number of annotated differentially expressed genes associated with each GO term is indicated on the horizontal axis. The 20 most enriched KEGG pathways for **(C)** the upregulated differentially expressed mRNA and **(D)** the downregulated differentially expressed mRNA. The size of the symbol represents the number of genes, and the colors represent the *p*-value.

DAVID (a database of annotation, visualization, and integrated discovery) was applied to perform GO and KEGG pathway analyses, which was carried out to figure out the function of DE mRNAs. The enrichment results of GO indicated that the most abundant biological processes of upregulated mRNA include primarily cellular process or metabolic process and cellular metabolic process; the most significant enriched cellular component was the cell, cell part, and intracellular; the most plentiful molecular function was binding and protein binding (as shown in Figure 5A). The most abundant biological processes of the downregulated mRNAs mainly include metabolic processes, cellular metabolic process, and cellular macromolecule metabolic processes; the most significantly enriched cellular components include intracellular, intracellular part, and organelle; the most enriched molecular functions consist of combinations of organic cyclic and heterocyclic compounds (Figure 5B). Moreover, the results of the KEGG analysis revealed that upregulated mRNAs were associated with endometrial cancer, colorectal cancer, and adherens junctions (Figure 5C). In contrast, downregulated mRNAs were involved in base excision repair and MAPK signaling pathways (Figure 5D).

### Functional Analysis of Differentially Expressed lncRNAs

**FIGURE 6:**
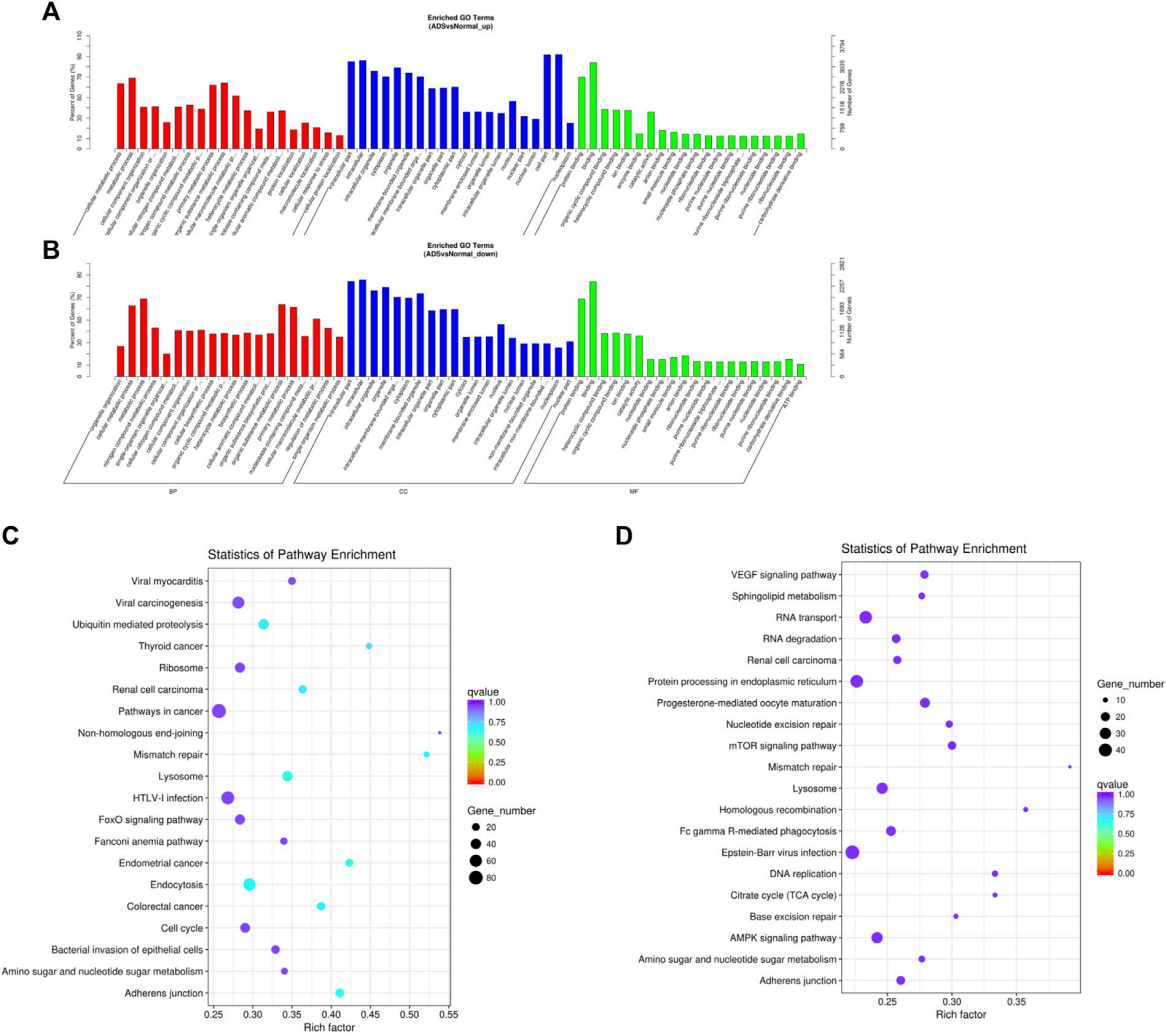
Comparison of functional annotations of the target gene of upregulated and downregulated differentially expressed lncRNAs. The 20 most enriched Gene Ontology (GO) terms for the parental genes of **(A)** the upregulated differentially expressed lncRNA and **(B)** the downregulated differentially expressed lncRNA. **(B)** Twenty most enriched KEGG pathways for **(C)** the upregulated differentially expressed lncRNA and **(D)** the downregulated differentially expressed lncRNA. The enriched GO terms are on the vertical axis, and the number of annotated target genes in each GO term is indicated on the horizontal axis. The size of the symbol represents the number of genes, and the colors represent the *p*-value.

Since lncRNAs have been reported to achieve the function by modulating mRNAs, we performed an extra-screen for DE lncRNAs associated with mRNAs. In brief, the coexpressed mRNAs of each DE lncRNA were identified by the correlation with protein-coding gene expression, which was used for functional enrichment analysis. Figure 6A–C displays the 20 most significantly enriched GO terms and the KEGG pathway in the upregulated lncRNAs; conversely, Figure 6B–D shows the 20 most enriched GO terms and the KEGG pathway in the downregulated lncRNAs. Enrichment analysis of the KEGG pathway indicated that the DE lncRNAs were associated with a multitude of molecular pathways, including endocytosis, lysosomes, adhesion junctions, mismatch repair, ubiquitin mediated proteolysis, and the AMPK signaling pathway.

### lncRNA–mRNA Coexpression Networks

**FIGURE 7:**
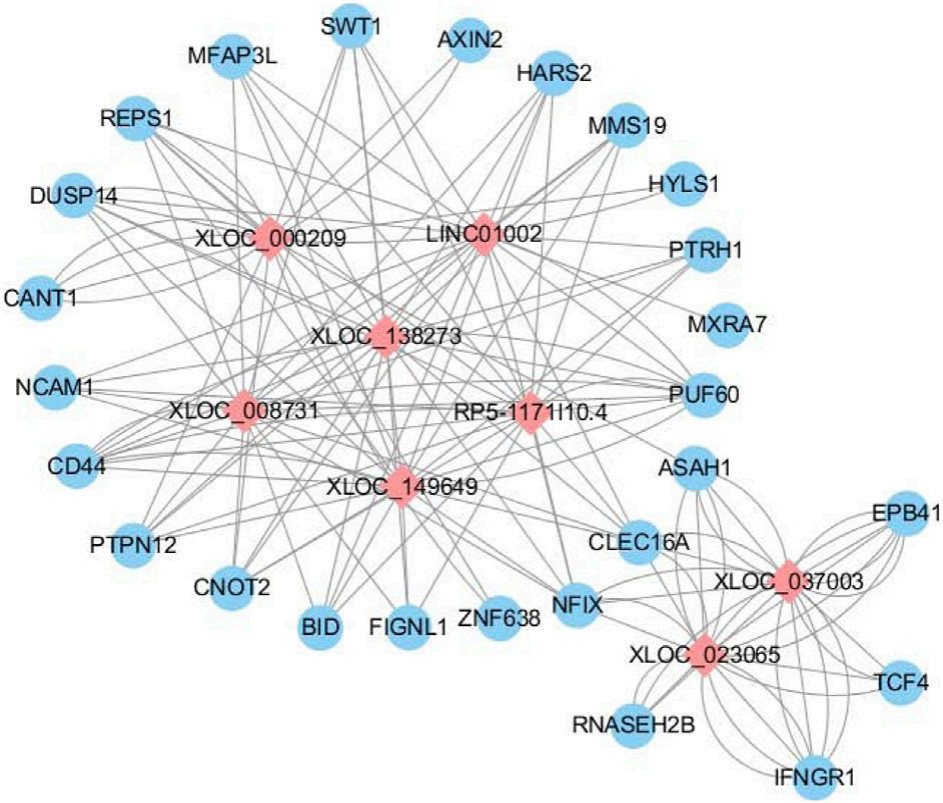
LncRNA–mRNA coexpression network. Differentially expressed lncRNA–mRNA regulatory networks consist of eight lncRNAs and 26 mRNAs. The blue circles represent mRNAs, and the red diamond represents lncRNAs. The Pearson correlation coefficient was limited to an absolute value > 0.95 and *p*-value <0.001.

We were able to anticipate the target gene of lncRNA through intersample coexpression analysis of lncRNA and mRNA, explore the synergistic effect of lncRNA and its differential expression targets, and identify the pairing that may be relevant to the pathogenesis of ADS. Ultimately, eight interested lncRNAs were generated in the analysis, and a lncRNA-mRNA coexpression network was constructed for visualization (Figure 7).

### Validation by Quantitative RT-PCR

When screening genes for PCR validation, the following three factors were considered: 1. high fold of expression difference between samples (log 2 (fold change)); 2. high gene expression (FPKM); and 3. relatively high gene sequencing depth readcount. Upon comprehensive analysis of the RNA-seq results, three DE mRNAs (HK1, CD44, and NFIX) and DE lncRNAs (XLOC_005,209, LINC01002, and XLOC_03,374) were selected for performing qRT-PCR to validate their expression levels further.

**FIGURE 8:**
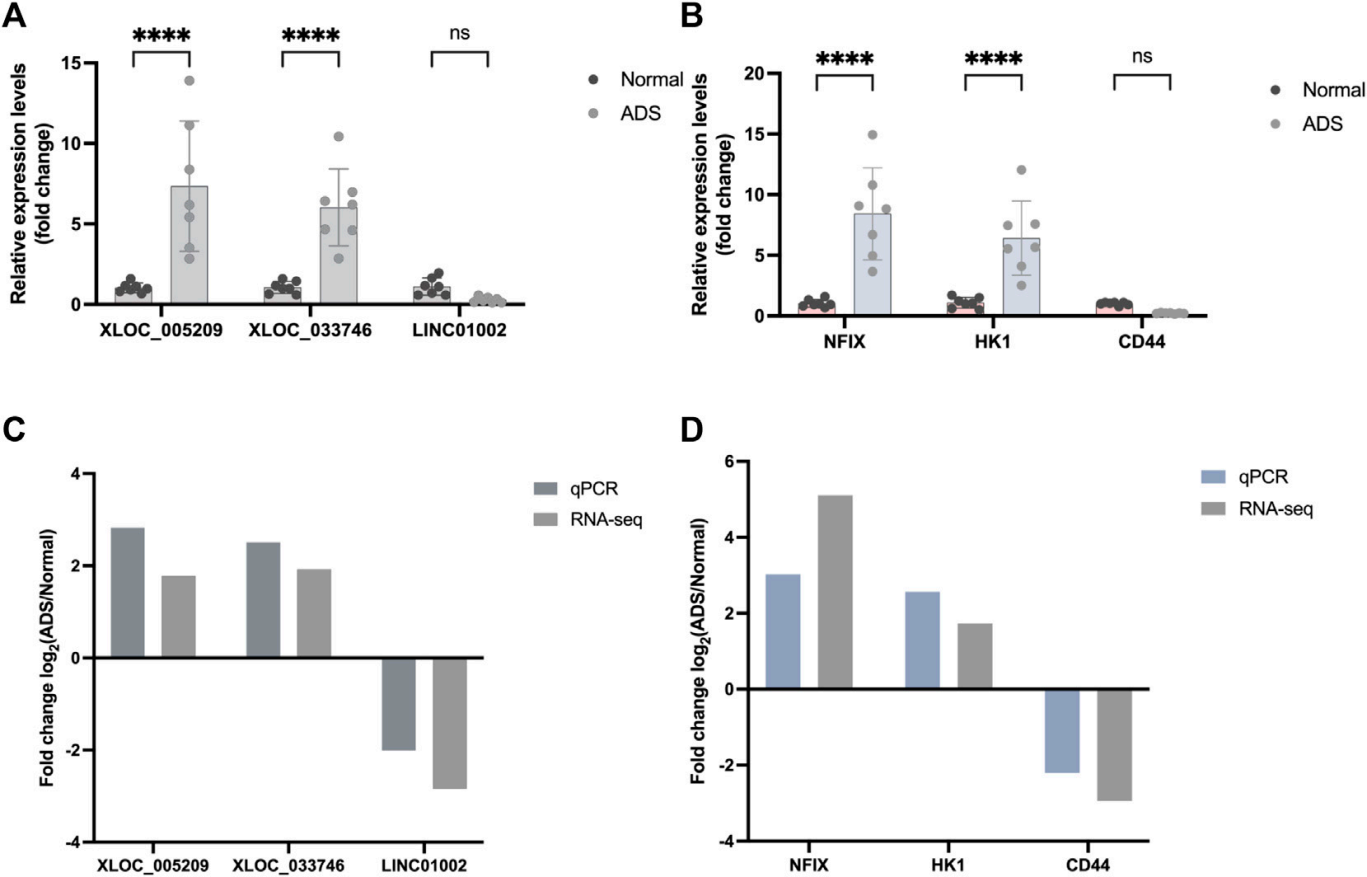
Validation for the expression of significant transcripts by quantitative RT-PCR. **(A)** Relative expression levels of qRT-PCR validation of three lncRNAs (*XLOC_005209, XLOC_033746*, and *LINC01002*) and **(B)** three mRNAs (*HK1, NFIX*, and *CD44*) are shown comparing ADS and normal groups. **(C–D)** Comparing qPCR results and RNA-seq data reveals a good correlation between such two methods. The heights of the columns represent the fold changes (log2 transformed) computed from the qPCR and RNA-seq data. Data are presented as mean ± SD, n = 7. * *p*-value < 0.05, ** *p*-value < 0.01, *** *p*-value < 0.001, and **** *p*-value <0.0001.

Eventually, the qRT-PCR data results showed consistency with the RNA-seq results (Figure 8), which further confirmed the reliability of the RNA-seq data.

### PPI Network

**FIGURE 9:**
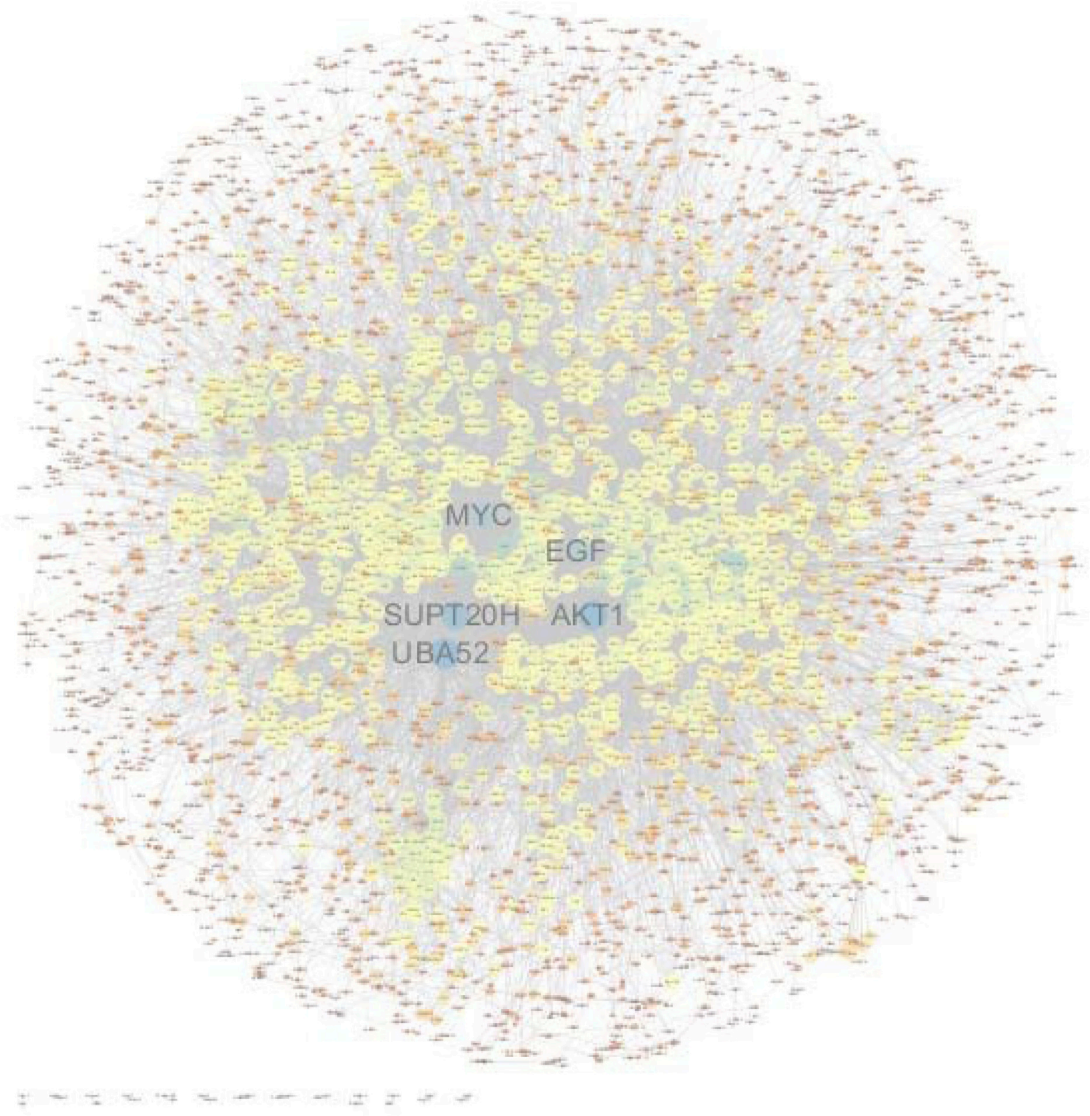
Protein–protein interaction network of significant differentially expressed genes. PPI nodes with relatively high connectivity include *UBA52*, *AKT1*, *SUPT20H*, *RPL19*, *EGF*, and *MYC*. The size of a node in a PPI map is proportional to the degree of the node.

In order to construct a visual network map, interaction relationships for the list of differential genes were extracted from the STRING protein interaction database (https://www.string-db.org/). The network data files were imported directly into Cytoscape software for visual editing. The size of a node in a PPI map is proportional to the degree of the node. Among them, PPI nodes with relatively high connectivity include *UBA52*, *AKT1*, *SUPT20H*, *RPL19*, *EGF*, and *MYC* (Figure 9).

## Discussion

ADS is a progressive, relentless, and special complex three-dimensional deformity of the spine (Kelly et al., 2020), characterized by occurrence in the lumbar spine with occasional compensatory thoracic curvature, rotation usually limited to the apex of the deformity, which has the potential to cause radiculopathy or spinal stenosis (Farfan, 1980; Bradford et al., 1999; Murata et al., 2002; Ploumis et al., 2007), and spondylolisthesis (Cho et al., 2014). The pathogenesis of ADS is similar to the starting point of degenerative spinal disease, namely, age-related disc degeneration (Kelly et al., 2020). The universally accepted theory is that age-related disc degeneration occurs in the general population, which is characterized by relatively symmetrical spinal degeneration without the onset of deformities. However, what confuses us is why some geriatric populations suffer from ADS? While degenerative processes are seen in a vast majority of the population with normal aging, what varies are the mechanical, nutritional, and inherited factors that can lead to more rapid progression, potentially resulting in significant pathology. In 2006, Kobayashi et al. (2006) found that osteophytes on the lateral endplate exceeding 5 mm or asymmetrical inclination of the IVD space exceeding 3 mm are essential risks factors for ADS. The asymmetric degenerative changes will result in a progressive imbalance of axial load. In contrast, an accelerating axial rotation will stretch the surrounding ligaments, which could aggravate degenerative changes and produce subsequent deformities, such as spinal instability, scoliosis, and/or kyphosis (Benner and Ehni, 1979). Therefore, ADS can be considered as the result of asymmetric degeneration and progressive coupling of the IVD and facet joint complex (Ploumis et al., 2007). The IVD is composed of three anatomical components: the central gelatinous NP, the outer annulus fibrosus (AF), and the cartilaginous endplate (CEP) that anchor onto the vertebral body. The NP is the core of the IVD and is surrounded by a lamella of AF. NP cells play a crucial role in maintaining the integrity of intervertebral discs *via* producing extracellular matrix (ECM) components, such as aggrecan alongside type II and type X collagen (Roughley, 2004). A growing body of evidence now suggests that aberrant NP cell functions, including altered cell proliferation, apoptosis, ECM production/degradation, and cytokine secretion, are key to IDD pathogenesis. It has been suggested that nucleus pulposus (NP) cells can activate the immune response once the blood-NP barrier is damaged, which is a crucial factor of IDD degeneration and can result in multiple pathological processes (Bridgen et al., 2017). Thus, WB samples can reflect the microenvironment and cytokines, which might be a breakthrough in discovering the pathogenesis of ADS.

According to reports, lncRNAs play an essential role in a wide-range of functional bioactivities. In recent years, many scholars have performed RNA sequencing on IDD and constructed a differential expression profile of RNA, which confirms that lncRNA plays a vital role in the development of IDD. A recent genetic study revealed a correlation between *COL2A1* polymorphism and ADS in Korean patients, suggesting a genetic component but failing to claim that this single nucleotide polymorphism (SNP) is a solitary genetic factor associated with ADS (Hwang et al., 2014). The significance of that study lies in the fact that it is the first study that the genetic factor should be considered as one of the multiple factors related to ADS, despite additional studies being warranted. Although several studies on molecular levels have addressed spinal degeneration, no such study has yet addressed the etiology of the ADS. Currently, considering that the molecular mechanisms of ADS are still poorly understood, few studies have focused on the differential RNA expression profile in ADS. Therefore, a comprehensive analysis of the DE lncRNAs and mRNAs and the identification of the candidate genes associated with ADS development may potentially be used to identify individuals at risk.

RNA-seq, a high-throughput technology, could provide a comprehensive view of the entire transcriptome, including subtype and gene fusion detection, gene expression profiling, targeted sequencing, and single-cell analysis (Hrdlickova et al., 2017). RNA-seq could facilitate the identification of novel genes, allele-specific expression, fusion genes, disease-associated single nucleotide polymorphisms (SNPs), post-transcriptional modifications, noncoding RNA (ncRNA), and differential gene expression between different groups or treatments (Byron et al., 2016). Adopting a novel RNA-seq analysis technique, this study demonstrated that the expression of lncRNAs and mRNAs in ADS patients differed from healthy patients. A total of 230 lncRNAs (147 upregulated and 83 downregulated) and 3,175 mRNAs (1,651 upregulated and 1,524 downregulated) were differentially expressed between ADS patients and healthy volunteers. *LNC_000044* and *ENST00000424684.2* were the most upregulated and downregulated lncRNAs. *EPB41* and *MYADM* were the most upregulated and downregulated mRNAs.

With the aim of better understanding the regulatory role of DE lncRNAs and the function of DE mRNAs, this study revealed differentially expressed genes associated with the WB samples of ADS and normal group through GO and KEGG pathway analysis. The results show that a substantial number of DE lncRNAs and mRNAs were discovered to be associated with inflammation, autophagy (mitochondrial autophagy, endothelial autophagy, and cellular autophagy), apoptosis, and angiogenesis, which are consistent with the recognized pathogenesis of ADS. Asymmetric disc degeneration is a multifactorial process, including mechanical stress, oxidative stress, aging, inflammation, genetic factors, the biological changes of IVD cells, extracellular matrix (ECM) degeneration, etc. (Hwang et al., 2016; Liu et al., 2016; Chen et al., 2017).

The most distinguishing feature of lncRNAs is the lack of ability to encode proteins, which means it is necessary to explain the biological function in other ways. For each DE lncRNA, the role of the lncRNA was inferred from the function of the corresponding mRNA by screening its corresponding coexpressed coding gene and finding the associated RNA-mRNA pair. In this study, we have selected eight interested lncRNAs to interact with 26 mRNAs, which performed lncRNA-mRNA coexpression network analysis. *PTPN12* plays a role as a modulator of hypoxia-induced AMPK activation and endothelial autophagy to facilitate angiogenesis, whereas endothelial autophagy is the prerequisite for angiogenesis (Chandel et al., 2021). Meanwhile, in the GO results, we found that AKT1 is also involved in cell migration and germinal angiogenesis. However, the IVD is a special organ without vascular and immune privileges (Tabana et al., 2016). Previous studies have shown that the development of disc degeneration is associated with angiogenesis (Ma et al., 2015). The degradation of ECM leads to the migration of endothelial cells, which in turn results in the formation of new blood vessels. The formation of new blood vessels exposes the NP to the immune system, which causes an immune response that leads to degenerative disease (Sun et al., 2013). Endothelial autophagy and angiogenesis may play a role in asymmetric disc degeneration of ADS, although more research is necessary to elucidate the underlying mechanisms in the future.

**FIGURE 10:**
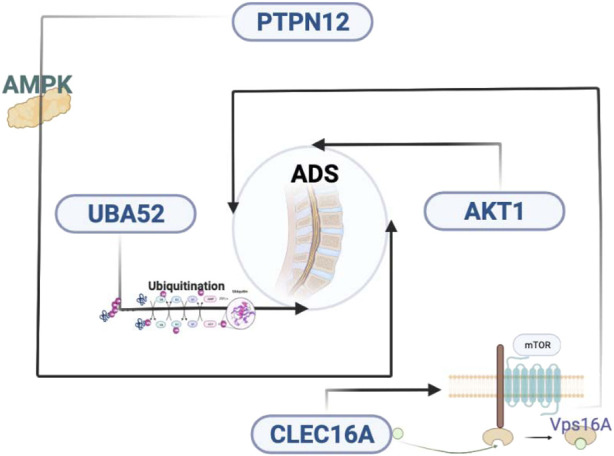
Key genes and molecular mechanisms that may be related to ADS. ADS, adult degenerative scoliosis; lncRNAs, long noncoding RNAs; mRNAs, messenger RNAs; RNA-seq, RNA sequencing; PPI, protein–protein interaction; NP: nucleus pulposus; IDD, intervertebral disc degeneration; qRT-PCR: quantitative RT-PCR; IVD, intervertebral disc; DE, differentially expressed; BMI, body mass index; PBS, phosphate-buffered saline; FPKM, fragments per kilobase of exon model per mapped reads; CC, cellular composition; MF, molecular function; BP, biological process; KEGG, Kyoto Encyclopedia of Genes and Genomes; FDR, false discovery rate; SNPs, single nucleotide polymorphisms; AF, annulus fibrosus; CEP, cartilaginous endplate; ECM, extracellular matrix; CEP, C-terminal elongation protein.

Subcellular localization studies have shown that *CLEC16A*, a membrane-associated endosomal protein, interacts with the E3 ubiquitin ligase Nrdp1, which is discovered in cytoplasmic vesicles and the Golgi apparatus. The deletion of *CLEC16A* induces the increase of Nrdp1 target Parkin, which is a principal regulator of mitochondrial autophagy (Soleimanpour et al., 2014). These findings suggest that Golgi-associated *CLEC16A* negatively regulates autophagy by modulating mTOR activity, as well as binding to Vps16A, a subunit of the class C Vps-HOPS complex, which could regulate receptor expression through autophagy (Tam et al., 2017; Pandey et al., 2019). Notably, *UBA52* had the highest connectivity in the PPI network in our study. *UBA52*, ubiquitin A-52 residue ribosomal protein fusion product 1, known as a protein-coding gene, encodes a protein composed of N-terminal ubiquitin and C-terminal ribosomal protein L40, a C-terminal elongation protein (CEP) (Lund et al., 1985). *Ubiquitin* is a highly conserved nuclear and cytoplasmic protein which is also involved in the maintenance of chromatin structure, regulation of gene expression, and stress responses. *UBA52* regulates the ubiquitination of ribosomes, while knockdown of *UBA52* always induces cell cycle arrest (Kobayashi et al., 2016). Ubiquitination can modulate the formation and nucleation of autophagosomes, which means that ubiquitination can control the autophagic process in response to various stress conditions. Autophagy plays a critical role in maintaining normal physiological processes, and dysregulation of ubiquitin-mediated autophagy has been associated with many diseases. A potential link may exist between ADS and disorders of the ubiquitin-mediated autophagic pathway (as shown in Figure 10).

To the best of our knowledge, this is the first report to conduct a genetic study on lncRNA-mRNA differential expression profiling performed on WB samples derived from ADS patients. Notably, the coexpression network of coding–noncoding genes provides valuable insights into the pathogenesis of ADS. Nowadays, the number of identified lncRNAs is multiplying. Therefore, further studies are necessary to explore their molecular and biological functions. Moreover, this study suffers from the following limitations. First, the samples that identified the cellular origin and tissue expression patterns of lncRNA were obtained from the WB of the ADS, which exclusively reflects local variations. Second, the sample size is relatively insufficient, which may limit the validity of the results. Third, the results were exclusively derived from bioinformatics analysis and high-throughput sequencing analysis, but without any animal experiments to further confirm these results. Furthermore, all the participants we enrolled in this study were Han Chinese from China. However, it is well known that ethnicity is also a factor affecting gene expression (Li et al., 2017). Since no other studies concerning the ADS have yet been reported, more studies will be needed to confirm the possible role of DE genes in the development of ADS.

## Conclusion

RNA-seq analysis provides a novel paradigm for investigating dysregulated lncRNAs and mRNAs. The differentially expressed genes may be involved in the regulation of the occurrence and development of ADS. This study provides a crucial biological basis and reference for exploring molecular markers or new gene targets for the diagnosis and treatment of adult degenerative scoliosis.

## Data Availability

The datasets presented in this study can be found in online repositories. The names of the repository/repositories and accession number(s) can be found below: GSE209825.
